# ZrBr_4_-Mediated Phase Engineering in CsPbBr_3_ for Enhanced Operational Stability of White-Light-Emitting Diodes

**DOI:** 10.3390/nano15090674

**Published:** 2025-04-28

**Authors:** Muhammad Amin Padhiar, Yongqiang Ji, Jing Wang, Noor Zamin Khan, Mengji Xiong, Shuxin Wang

**Affiliations:** 1 School of Intelligent Manufacturing, Shanghai Zhongqiao Vocational and Technical University, Shanghai 201205, China; amin.padhiar@gzhu.edu.cn (M.A.P.); jennywang_0611@163.com (J.W.); xmj1631632025@163.com (M.X.); 2 School of Physics and Materials Science, Guangzhou University, Guangzhou 510006, China; noorzamin@gzhu.edu.cn; 3 Institute of Physics, Henan Academy of Sciences, Zhengzhou 450046, China

**Keywords:** all-inorganic perovskite nanocrystals, phase transformation, radiative recombination, white-light-emitting diodes (WLEDs), luminous efficiency, operational stability

## Abstract

The persistent operational instability of all-inorganic cesium lead halide (CsPbX_3_) perovskite nanocrystals (NCs) has hindered their integration into white-light-emitting diodes (WLEDs). This study introduces a transformative approach by engineering a phase transition from CsPbBr_3_ NCs to zirconium bromide (ZrBr_4_)-stabilized hexagonal nanocomposites (HNs) through a modified hot-injection synthesis. Structural analyses revealed that the ZrBr_4_-mediated phase transformation induced a structurally ordered lattice with minimized defects, significantly enhancing charge carrier confinement and radiative recombination efficiency. The resulting HNs achieved an exceptional photoluminescence quantum yield (PLQY) of 92%, prolonged emission lifetimes, and suppressed nonradiative decay, attributed to effective surface passivation. The WLEDs with HNs enabled a breakthrough luminous efficiency of 158 lm/W and a record color rendering index (CRI) of 98, outperforming conventional CsPbX_3_-based devices. The WLEDs exhibited robust thermal stability, retaining over 80% of initial emission intensity at 100 °C, and demonstrated exceptional operational stability with negligible PL degradation during 50 h of continuous operation at 100 mA. Commission Internationale de l’Éclairage (CIE) coordinates of (0.35, 0.32) validated pure white-light emission with high chromatic fidelity. This work establishes ZrBr_4_-mediated HNs as a paradigm-shifting material platform, addressing critical stability and efficiency challenges in perovskite optoelectronics and paving the way for next-generation, high-performance lighting solutions.

## 1. Introduction

All inorganic cesium lead halide (CsPbX_3_) perovskite nanocrystal (NC)-based white-light-emitting diodes (WLEDs) have revolutionized lighting and display technologies due to their energy efficiency. However, achieving high-performance WLEDs with stable color output under operational conditions remains a significant challenge. CsPbX_3_ NCs demonstrate unique structural, optical, and electrical characteristics and have emerged as promising candidates for WLEDs, owing to their exceptional photoluminescence quantum yields (PLQYs > 95%), tunable bandgaps, and narrow emission spectra [1,2,3]. Despite these advantages, the commercialization of perovskite-based WLEDs is hindered by their intrinsic instability under thermal, moisture, and photolytic stress, which accelerates ion migration and phase segregation, leading to rapid degradation of optoelectronic performance [4,5,6]. Advancements of halide NCs in optoelectronics have seen rapid progress in recent years [7,8,9,10]. Nevertheless, substantial obstacles continue to impede the transition of halide NCs from the laboratory to widespread commercial deployment of WLEDs due to stability issues of the poor material quality [11,12,13,14]. Recently, surface passivation became an immediate solution to address these stability issues [15]. For instance, organic molecules and ligand engineering have been shown to effectively enhance the stability of halide NCs [16]. However, applying such a coating can reduce the conductivity of halide NCs films, creating an undesirable electronic energy barrier that hinders efficient charge injection or extraction in HP-based optoelectronic devices [17]. Besides surface passivation, constructing heterostructures has also been found to improve the stability [18,19,20]. However, the large lattice mismatch between the core and shell materials often leads to interfacial defects. Among the various options, silica is the most commonly used coating material due to its wide compatibility with core materials and its ability to provide the necessary robustness to withstand external disturbances [21]. The relatively thick silica shell can hinder light extraction, thereby limiting overall device performance [22]. In contrast, metal oxide coatings exhibit remarkable chemical stability encapsulating halide NCs in these materials, effectively protecting them from environmental degradation and significantly enhances their long-term stability [23]. The conventional coating methods often prove ineffective due to the highly ionic nature of halide NCs [24]. Besides the above-mentioned strategies, doping and ion substation into the lattice is another promising method to enhance the halide NCs material properties [25]. Various groups performed successful substitution ions in halide NCs through doping [26,27,28]. Although dopants can provide several advantages, they can also negatively affect the characteristics by introducing additional defect states, increasing nonradiative recombination, or inducing structural distortions [29].

In this context, phase engineering and morphological transformation showed an effective approach to modify the crystal lattice and electronic structure, resulting in enhanced charge carrier mobility in halide NCs [30]. Zhao and coworkers performed shape-controlled synthesis of CsPbBr_3_ nanorods (NRs) with pure blue light emission. The uniform morphology was achieved by precisely controlling the synthesis conditions [31]. Zhang et al. developed an in situ transformation method to fabricate dual-phase Cu/CsPbBr_3_-Cs_4_PbBr_6_ NCs. These NCs exhibit a 4.2-fold enhancement in photocatalytic efficiency for CO_2_ reduction, which is attributed to a 303% increase in the electron consumption rate compared to pristine CsPbBr_3_ NCs [32]. Moreover, we demonstrated the successful phase transformation of lead-free Bi^3+^/Mn^2+^ co-doped Cs_2_NaInCl_6_ NCs, converting 0D NCs into 2D nanosheets (NSs). The resulting 2D NSs exhibited a PLQY of 73%, nearly double that of the pristine 0D NCs [33]. Ivan Ornelas-Cruz and colleagues used density functional theory to study the cubic-to-hexagonal phase transition in nine metal halide compounds of the form APbX_3,_ where the A-site is occupied by HC(NH_2_)_2_^+^, CH_3_NH_3_^+^, or Cs^+^ and the X-site by I^−^, Br^−^, or Cl^−^ [34]. Recently, Zhang et al. transformed Bi-doped Cs_2_Ag_x_Na_1-x_lnCl_6_ NCs into NPSs, achieving PLQY close to unity [35]. Chan et al. demonstrated the mechanism behind the formation of CsPbBr_3_ superstructures (SCs) during the hot injection synthesis of CsPbBr_3_ NCs. The excess, unreacted PbBr_2_ in the reaction mixture acts as a “glue” to link the individual CsPbBr_3_ NCs together, forming the SCs [36]. In another study, Ghorai et.al studied phase segregation in CsPbI_1.5_Br_1.5_ NCs under continuous 325 nm laser illumination using PL spectroscopy and real-time imaging [37]. Although numerous researchers have focused on phase engineering in halide nanocrystals, the morphological transformation for most elements remains largely unexplored. This is primarily because controlling shape evolution during synthesis demands precise adjustments of parameters such as temperature, precursor concentration, and the ligand environment. Based on previous studies, investigating these phenomena is crucial for enhancing device performance for future applications. Herein, we demonstrated a co-rational strategy to stabilize the hexagonal phase of CsPbBr_3_ NCs by introducing ZrBr_4_ as a phase-directing mediator. While the cubic phase of CsPbBr_3_ is commonly reported in colloidal syntheses, the hexagonal phase with surface passivation offers distinct advantages for optoelectronic applications, including enhanced thermal stability and anisotropic charge transport properties.

## 2. Results and Discussion

### 2.1. Morphological Analysis

**Figure 1:**
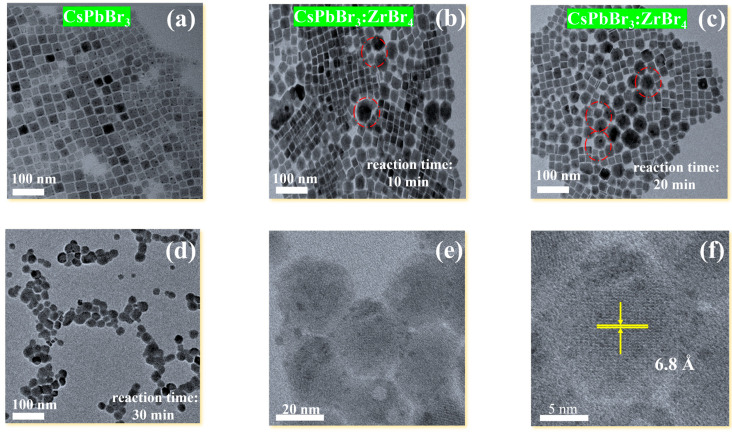
Morphological analysis. (**a**) Initial TEM image of CsPbBr_3_ NCs. (**b**,**c**) TEM image of NCs after 10 and 20 min reaction time. (**d**) HNs completely formed after 30 min reaction time. (**e**,**f**) HRTEM images of HNs.

In this manuscript, we study the phase transformation of CsPbBr_3_ NCs mediated by *_x_*ZrBr_4_, where *x* represents PbBr_2_:ZrBr_4_ molar ratios (*x* = 0.25, 0.50, 0.1, and 0.2 respectively) with a modified hot injection method. The detailed synthesis methodology and specific feed ratios are detailed in the Supplementary Information (Table S1). To investigate the phase kinetics and transformation, we conducted transmission electron microscopy (TEM) analysis. Figure 1a presents TEM images of pure CsPbBr_3_ NCs, while Figure 1b–d represents CsPbBr_3_:*_x_*ZrBr_4_ NCs (*x* = 0.1). As shown in Figure 1b–d by systematically varying the reaction time to (10 min, 20 min, and 30 min), we observed a progressive transformation from a mixed cubic-hexagonal phase to phase-pure hexagonal nanocomposites (HNs). At 10 min, the partial hexagonal phase (~40%) coexists with residual cubic domains (Figure 1b). The limited time allowed the initial Zr^4+^ incorporation but insufficient lattice rearrangement. Conversely, the 20 min reaction time hexagonal phase dominated (~70%), as extended heating enhanced the Zr^4+^-mediated strain and suppressed cubic defects (Figure 1c).

**Figure 2:**
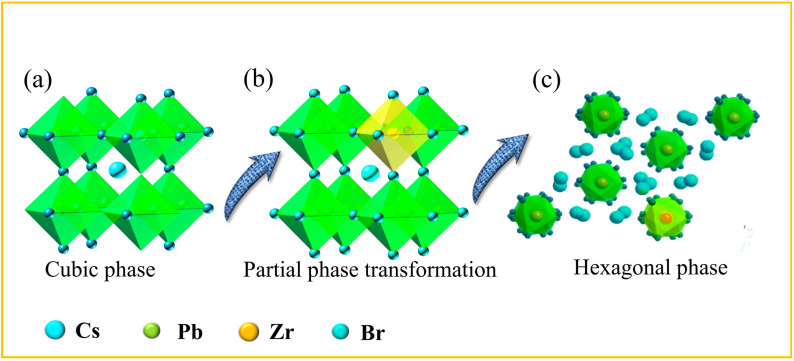
(**a**–**c**) Crystal structure of the proposed phase transformation from the NC HN process.

Upon 30 min reaction, time phase-pure HNs (~98%) were achieved, with Zr^4+^ occupying Pb^2+^ sites and stabilizing the hexagonal framework (Figure 1d) [38]. The phase transformation stages can be seen in the Supplementary Information (Table S2). The red-circled regions highlight the formation of interfacial defects and localized lattice distortions. These defects likely arise from Zr^4+^ ion substitution into the Pb^2+^ sites of the perovskite lattice, destabilizing the original cubic structure [36,37]. The presence of Br^−^ ions from ZrBr_4_ may also promote anion vacancies, further disrupting the crystal lattice. Similar ion substitution mechanisms have been observed in Zn- doped CsPbBr_3_ NCs [38,39]. Figure 1e,f show HRTEM with the average lattice size in this region being approximately 6.8 (Å) (110) plane. The schematic diagrams (Figure 2a–c) depict the proposed phase transformation process. Initially (Figure 2a), the pristine CsPbBr_3_ exhibited a cubic lattice structure with Pb^2+^ ions occupying the B-sites. Upon introducing Zr^4^⁺ ions, substitution at Pb^2+^ sites occurred, leading to lattice defects and destabilization. Concurrently, Br^−^ ions from ZrBr_4_ migrated to the surface, promoting anion vacancies and facilitating nanoparticle (NP) nucleation. Ultimately (Figure 2b,c), this progression resulted in the complete decomposition of CsPbBr_3_ NCs into isolated CsPbBr_3_:*_x_*ZrBr_4_ HNs and amorphous regions. This transformation mechanism is similar to the Schottky defect formation observed in halide perovskites [40]. The strong Lewis acidity and its ability to coordinate with Br- ions alter the local surface energy and lattice strain. This modulation directs anisotropic growth and favors the reorganization of the crystal lattice into a hexagonal structure rather than the typical cubic phase. Additionally, Zr helps in passivating surface defects, further stabilizing the hexagonal phase and enhancing charge carrier mobility, which is crucial for improved optoelectronic performance.

**Figure 3:**
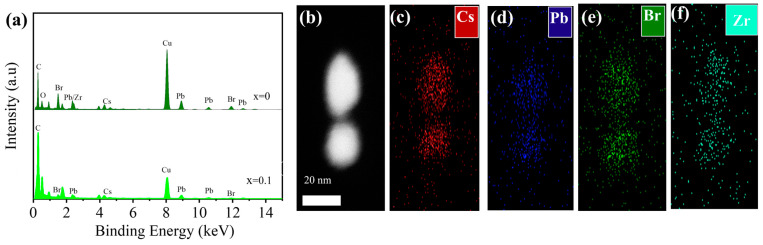
(**a**) EDS graphs of CsPbBr_3_:*_x_*ZrBr_4_ where (*x* = 0 and 0.1). (**b**) HAADF-TEM image of CsPbBr_3_:*_x_*ZrBr_4_ where (*x* = 0.1). (**c**–**f**) Elemental mappings of Cs, Pb, Br, and Zr elements in the CsPbBr_3_:*_x_*ZrBr_4_ where (*x* = 0.1) HNs.

To understand the elemental compositions, we performed energy-dispersive X-ray spectroscopy (EDS) analysis of CsPbBr_3_ and CsPbBr_3_:*_x_*ZrBr_4_ (*x* = 0.1), as shown in Figure 3a, revealing that the Cs, Pb, and Br peaks corresponded to standard values for CsPbBr_3_, suggesting the presence of Pb^2+^ oxidation states. The detection of Zr and O elements indicated potential inclusion and surface adsorption. Notably, CsPbBr_3_ with Zr^+^ cations enhanced compositional stability. Similar observations were studied with ZnBr^2+^ ions for improved stability [41]. Elemental mapping of CsPbBr_3_:*_x_*ZrBr_4_ (*x* = 0.1) in Figure 3b–f revealed a uniform distribution of Cs (red), Pb (blue), Br (green), and Zr (cyan) throughout the sample, indicating minimal segregation during the phase transition. This uniformity suggests that the incorporation of Zr^4+^ ions into the CsPbBr_3_ lattice likely occurs through substitutional doping, where Zr^4+^ replaces Pb^2+^ ions. Such substitution introduces lattice distortions, which can lower the energy barrier for phase transitions, facilitating the transformation process. This mechanism aligns with observations in similar perovskite systems, where dopant ions influenced the structural stability and phase behavior [42].

**Figure 4:**
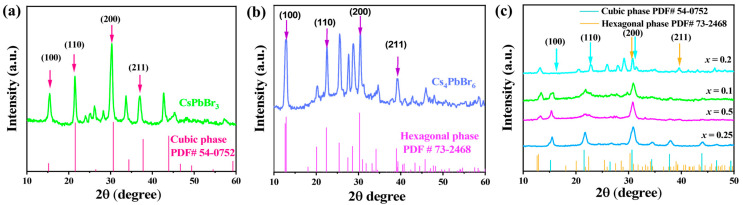
(**a**–**c**) XRD patterns of pristine CsPbBr_3_ NCs and transformed phase of CsPbBr_3_:*_x_*ZrBr_4_ where (*x* = 0.25, 0.5, 0.1, and 0.2) HNs.

Figure 4a–c present the X-ray diffraction (XRD) patterns comparing the cubic and hexagonal phases of CsPbBr_3_. The pristine CsPbBr_3_ exhibited a cubic phase, aligning with the standard (PDF 54-0752), characterized by dominant peaks at (100), (110), (200), and (211) reflections, indicative of its high-symmetry structure with sharp, narrow peaks. In contrast, the hexagonal phase, corresponding to (PDF 73-2468), displayed new peaks at lower angles, reflecting its lower symmetry lattice. The broader peaks observed suggest slight disorder or strain within the hexagonal phase. Figure 4c illustrates the XRD patterns for various PbBr:_2_ZrBr_4_ feed ratios ranging from (*x* = 0.25 to 0.2), respectively. At (*x* = 0.25), weak hexagonal peaks indicated the early stages of nucleation. As the ratio increased from (*x* = 0.50 to 0.1), hexagonal peaks became predominant, confirming a complete phase transformation. This suggests that higher concentrations of ZrBr_4_ (e.g., *x* = 0.2) facilitate the formation of the hexagonal phase, likely due to Zr doping enhancing lattice instability.

The substitution of Zr^4+^ (ionic radius 0.72 Å) for Pb^2+^ (ionic radius 0.76 Å) led to lattice contraction, destabilizing the cubic structure and promoting the formation of a more compact hexagonal lattice (CsPbBr_3_-H) [43,44]. The increasing intensity of the hexagonal phase XRD peaks at higher doping levels (x > 0.1) indicated its energetic favorability under these conditions.

**Figure 5:**
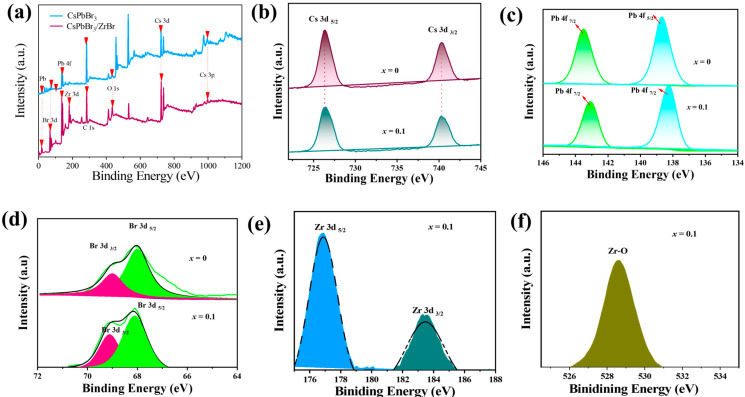
(**a**) XPS survey of CsPbBr_3_ NCs and CsPbBr3:*_x_*ZrBr_4_ HNS. High-resolution XPS of (**b**) Cs 3d, (**c**) Pb 4f, (**d**) Br 3d, (**e**) Zr 3d. (**f**) O 1s.

To further confirm the chemical composition of the CsPbBr_3_ and transformed phase of CsPbBr_3_:xZrBr_4_ (*x* = 0.1), X-ray photoelectron spectroscopy (XPS) analysis was conducted. As shown in Figure 5a, the XPS spectra of both samples clearly exhibited signals of Cs/Pb/Br/, while Zr was only present in the *x* = 0.1 sample. Figure 5b shows the XPS spectra of Cs elements. The binding energy of Cs 3d electrons in CsPbBr_3_ was located at 726.5 eV, whereas CsPbBr_3_:*_x_*ZrBr_4_ shifted to 726.03eV due to Zr incorporation, indicating slight peak shift in the lattice. From the XPS spectra in Figure 5c for Pb 4f, as well as Figure 5d for Br 3d, we observe that the Zr interaction led to a shift of their electron binding energies to higher energy peaks. This may be attributed to the distortion of the octahedral structure induced by Zr and access Br supply, affecting the bond lengths or bond angles of Br–Pb–Br, making it difficult for them to lose electrons. Hence, the XPS peaks of these elements shifted to higher energy. Figure 5e displays the XPS spectra of Zr 3d, confirming the successful integration of Zr ions. The presence of a Zr-O peak in Figure 5f suggests the formation of the Zr-O bond, indicating that Zr has been incorporated into HNs. This incorporation can influence the material properties and be beneficial in enhancing thermal stability or modifying electronic characteristics. For instance, in studies involving Zr-doped materials, the formation of Zr-O bonds has been associated with improved structural stability and altered electronic environments [45].

### 2.2. Optical Properties

**Figure 6:**
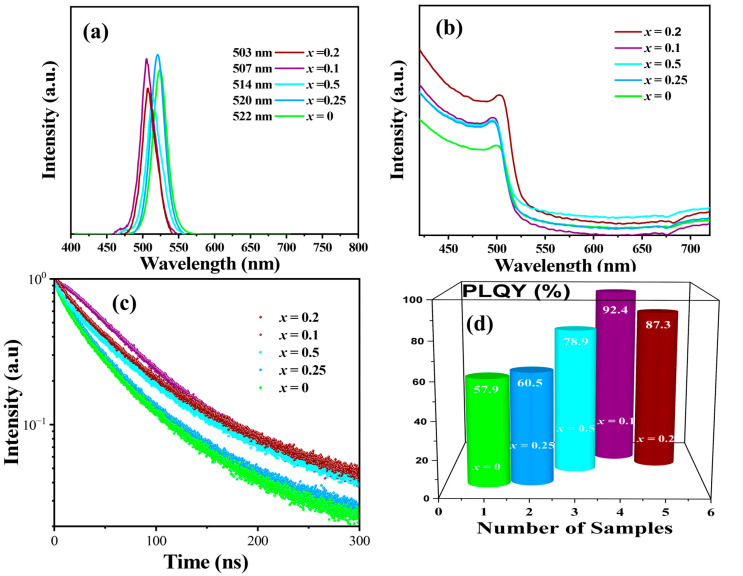
Optical characterization of CsPbBr_3_ NCs and CsPbBr_3_:*_x_*ZrBr_4_ HNS (*x* = 0.25, 0.5, 0.1, 0.2). (**a**) PL wavelengths. (**b**) Absorbance spectra. (**c**) TRPL curves. (**d**) PLQY of the corresponding samples.

To investigate the optical properties of pristine CsPbBr_3_ NCs and CsPbBr_3_:*_x_*ZrBr_4_ HNs with varying ZrBr_4_ feed ratios (*x* = 0.25, 0.5, 0.1, and 0.2), we conducted PL analysis, as shown in Figure 6a. The PL spectra of pristine CsPbBr_3_ NCs exhibited a peak centered around 522 nm (Figure 6a), indicating pure green emission.

During phase transition induced by increasing ZrBr_4_ feed ratios from (*x* = 0.25 to 0.1), a noticeable blue shift in PL emission occurred, with peaks shifting from 520 nm to 503 nm (Figure 6a). The PL intensity initially increased, reaching a maximum at (*x* = 0.1), but showed a slight decrease at (*x* = 0.2), with the emission remaining around 503 nm (Figure 6a). The absorption properties of CsPbBr_3_ NCs took varying concentrations to determine the optimal composition. The absorption spectrum of pristine CsPbBr_3_ NCs exhibited an absorption edge, as shown in Figure 6b. With increasing ZrBr_4_ concentration, a noticeable blue shift in the absorption edge was observed, moving to shorter wavelengths. This shift corresponded with the observed blue shift in PL emissions indicating consistent changes in the electronic structure of the NCs due to Zr incorporation. Figure 6c shows time-resolved (TRPL) analysis insights into the carrier recombination dynamics within Zr-mediated phase-transformed CsPbBr_3_ HNs. By fitting the PL decay curves with bi-exponential decay model Equations (S1) and (S2), the detailed TRPL values are as presented in Table S3 of the Supplementary Information. We could distinguish between trap-assisted (non-radiative) and radiative recombination processes. The fast decay component corresponded to trap-assisted recombination, while the slow decay component was associated with radiative recombination. The ZrBr_4_ charge dynamics into CsPbBr_3_ led to an enhanced radiative recombination efficiency, as indicated by an increased amplitude of the slow decay component. This enhancement resulted in a longer average PL lifetime, reflecting a PLQY, as shown in Figure 6d. The observed blue shift in PL emission and the variation in PLQY can be attributed to the substitution of Pb^2+^ ions, leading to lattice contraction and destabilization of the cubic structure [44]. Stability is a major concern of halide NCs, and their inherent instability, particularly at elevated temperatures, poses significant challenges for practical use. This thermal instability is primarily attributed to the low formation energy of halide NCs, which makes them susceptible to decomposition under heat [45,46,47]. Additionally, the organic ligands that stabilize halide NCs can detach or degrade when exposed to high temperatures, leading to NPs aggregation and loss of functionality [48]. Introducing ZrBr_4_ into CsPbBr_3_ halide NCs enhanced their thermal stability, primarily due to the structural phase transition from a cubic to a hexagonal lattice. These transitions can introduce structural stability, affecting the material’s performance under varying thermal conditions [49].

**Figure 7:**
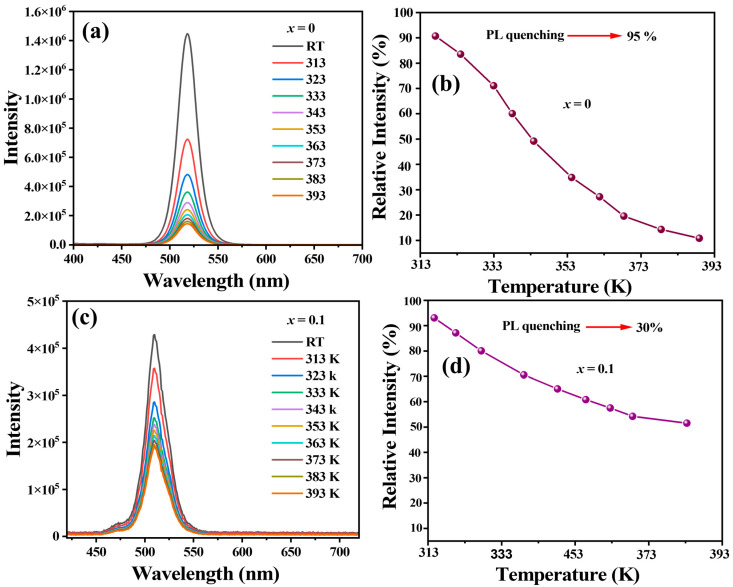
(**a**) Temperature-dependent PL analysis. (**a**,**c**) PL spectra of CsPbBr_3_ NCs and CsPbBr_3_:*_x_*ZrBr_4_ HNS (*x* = 0.1) recorded from RT to 393K; (**b**,**d**) the relative change in PL intensity showing PL quenching.

To evaluate their stability, we conducted a temperature-dependent PL analysis of pristine CsPbBr_3_ NCs and ZrBr_4_-mediated HNs (with *x* = 0.1) over a temperature range from room temperature (RT) to 393 K, as shown in Figure 7. As the temperature increased from RT to 393 K, the PL intensity of pristine CsPbBr_3_ NCs exhibited a significant decrease, indicative of thermal quenching, a common phenomenon where increased thermal energy enhances non-radiative recombination pathways, leading to reduced PL intensity. Specifically, the PL intensity diminished by approximately 60% at 373 K and further to 95% at 393 K compared to the initial intensity at RT, as seen in Figure 7a. In contrast, the ZrBr_4_-mediated HNs demonstrated enhanced thermal stability, as shown in Figure 7c. The PL intensity remained relatively stable up to 353 K, with only a minor reduction observed; this suggests robust structural stability and reduced electron–phonon coupling after ZrBr_4_ HN phase engineering. The relative PL intensives of the corresponding samples are shown in Figure 7b,d.

The improved thermal stability of the ZrBr_4_-mediated HNs can be attributed to the Zr^4+^ ions, which facilitate the formation of a hexagonal phase, enhancing the structural robustness of the material. This phase transition likely mitigates the formation of surface defects and suppresses non-radiative recombination pathways, thereby preserving PL intensity at elevated temperatures. The ZrBr_4_-mediated phase transformation in CsPbBr_3_ NCs to a HNs significantly enhanced thermal stability, maintaining higher PL intensity and consistent emission characteristics across a broad temperature range. Similarly, Yang et al. showed enhanced thermal stability in α-ZrP/CsPbBr_3_ composites [50]. This enhancement is crucial for the development of perovskite-based optoelectronic devices that operate reliably under varying thermal conditions.

### 2.3. Device Performance

Finally, taking advantage of enhanced PLQY and stability, we tested the operational performance of ZrBr_4_-mediated CsPbBr_3_ HNs. To construct the WLED, we combined green-emitting CsPbBr_3_:*x*ZrBr_4_ (*x* = 0.1) HNs with red-emitting CsPbBrI_2_ NCs and integrated them onto a 450 nm blue LED chip.

**Figure 8:**
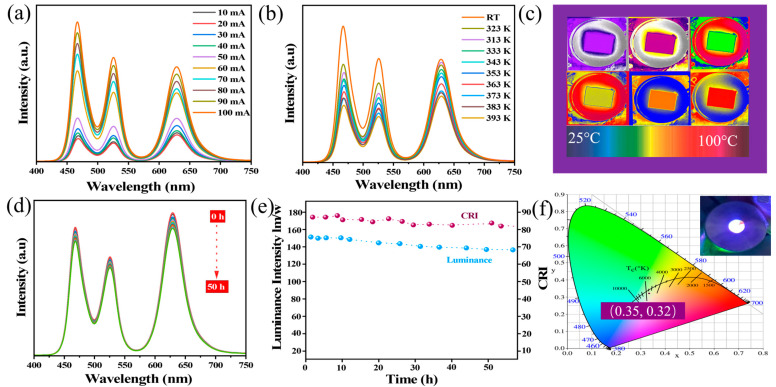
WLED operational analysis of CsPbBr_3_:*_x_*ZrBr_4_ HNS (*x* = 0.1). (**a**). PL intensities with variable currents from 10 mA to 100 mA. (**b**) PL intensities with variable temperatures from RT to 393 K. (**c**) WLED physical images taken under a thermal image camera. (**d**) WLED performance under a 100 mA current with respect to time from 0 to 50 h. (**e**) Luminance and CRI values. (**f**) CIE diagram with an inset of WLED.

The resulting device exhibited distinct emission peaks corresponding to the blue chip, green HNs, and red NCs, as shown in Figure 8. The WLED performance was tested under different current ratings (amperes), as shown in Figure 8a. The WLED exhibited PL intensity shifts upon varying the current from 10 mA to 100 mA, with the highest intensity recorded at 100 mA. WLEDs typically generate heat during prolonged operation, leading to potential thermal degradation. To assess this, we monitored the surface temperature and PL spectra of the WLED under different temperatures, as presented in Figure 8b,c. The surface temperature rose to approximately 100 °C. Despite this elevated temperature, the PL intensity of the WLED retained over 80% of its initial value. Moreover, continuous operation of the WLED was tested at a fixed room temperature (RT) and a 100 mA current. After 50 h of continuous operation, only minimal shifts in PL intensity were observed, as shown in Figure 8d. Notably, the WLED achieved a high luminous efficiency of 158 lm/W, with the color rendering index (CRI) reaching 98, as shown in Figure 8e. Table 1 shows compression of this work with some recent WLED devices lm/W efficiency. The CIE coordinates of the WLED, measured at (0.35, 0.32), are presented in Figure 8f, indicating pure white-phase light emission. This performance surpasses that of traditional CsPbX_3_ LEDs, which often exhibit lower luminous efficiencies and CRI values that decrease with increasing drive current. These findings underscore the enhanced thermal stability and optical performance of Zr-mediated CsPbBr_3_ HNs, positioning them as promising candidates for advanced optoelectronic applications, particularly in the development of efficient and stable WLEDs.

## 3. Conclusions

This study addresses the critical challenge of operational instability in CsPbBr_3_ halide NCs by developing a novel ZrBr_4_-mediated phase transformation strategy to synthesize HNs with enhanced structural and optoelectronic properties. The ZrBr_4_ integration induced a structurally ordered lattice, effectively passivating surface defects and suppressing nonradiative recombination pathways. This optimization yielded HNs with a remarkable PLQY of 92%, extended emission lifetimes, and exceptional thermal and operational stability, retaining over 80% of initial PL intensity at 100 °C and demonstrating negligible degradation under prolonged operation. The fabricated WLEDs achieved a luminous efficiency of 158 lm/W and a record CRI of 98, with CIE coordinates (0.35, 0.32) confirming pure white-light emission. These metrics surpass conventional CsPbX_3_-based LEDs, underscoring the efficacy of ZrBr_4_-mediated HNs in overcoming intrinsic limitations of traditional halide NCs.

## Figures and Tables

**Table 1:** The comparison among our devices’ previously reported WLEDs.

Color-Converting Materials	lm/W	References
CsPbBr_3_ QDs	63	[51]
CsPbBr_3_/Cs4PbBr_6_ composites	129.5	[52]
CsPbBr_3_/EVA /AlSiN_3_:Eu^2+^ phosphors	37.7	[53]
CsPbBr3@SiO_2_ QDs/ Red AgInZnS QDs	40.6	[54]
CsPBr_3_:ZrBr_4_ HNs	158	his work

## Data Availability

The results supporting this study are presented in the manuscript and the Supplementary Materials.

## Supplementary Materials

The following supporting information can be downloaded at: https://www.mdpi.com/article/10.3390/nano15090674/s1, Table S1. composition of each element during synthesis process. Table S2. The phase transformation stages. Table S3. Bi-exponential fitting results of the time resolved PL spectra of CsPbBr_3_:ZrBr_4_ (*x* = 0, 0.25, 0.5, 0.1 and 0.2) respectively.

## References

[1] Shi D., Adinolfi V., Comin R., Yuan M., Alarousu E., Buin A., Chen Y., Hoogland S., Rothenberger A., Katsiev K. (2015). Low Trap-State Density and Long Carrier Diffusion in Organolead Trihalide Perovskite Single Crystals. Science.

[2] Tian J., Xue Q., Yao Q., Li N., Brabec C.J., Yip H.-L. (2020). Inorganic Halide Perovskite Solar Cells: Progress and Challenges. Adv. Energy Mater..

[3] Chen D., Fang G., Chen X. (2017). Silica-Coated Mn-Doped CsPb(Cl/Br)_3_ Inorganic Perovskite Quantum Dots: Exciton-to-Mn Energy Transfer and Blue-Excitable Solid-State Lighting. ACS Appl. Mater. Interfaces.

[4] Li F., Xia Z., Pan C., Gong Y., Gu L., Liu Q., Zhang J.Z. (2018). High Br– Content CsPb(Cl_y_Br_1–y_)_3_ Perovskite Nanocrystals with Strong Mn^2+^ Emission Through Diverse Cation/Anion Exchange Engineering. ACS Appl. Mater. Interfaces.

[5] Gupta G.K., Kim I.J., Park Y., Kim M.K., Lee J.S. (2023). Inorganic Perovskite Quantum Dot-Mediated Photonic Multimodal Synapse. ACS Appl. Mater. Interfaces.

[6] Li M.-Z., Guo L.-C., Ding G.-L., Zhou K., Xiong Z.-Y., Han S.-T., Zhou Y. (2021). Inorganic Perovskite Quantum Dot-Based Strain Sensors for Data Storage and In-Sensor Computing. ACS Appl. Mater. Interfaces.

[7] Moyen E., Jun H., Kim H.-M., Jang J. (2018). Surface Engineering of Room Temperature-Grown Inorganic Perovskite Quantum Dots for Highly Efficient Inverted Light-Emitting Diodes. ACS Appl. Mater. Interfaces.

[8] Shen X., Sun C., Bai X., Zhang X., Wang Y., Wang Y., Song H., Yu W.W. (2018). Efficient and Stable CsPb(Br/I)_3_@Anthracene Composites for White Light-Emitting Devices. ACS Appl. Mater. Interfaces.

[9] Zhang X., Qian Y., Ling X., Wang Y., Zhang Y., Shi J., Shi Y., Yuan J., Ma W. (2020). α-CsPbBr_3_ Perovskite Quantum Dots for Application in Semitransparent Photovoltaics. ACS Appl. Mater. Interfaces.

[10] Gong R., Wang F., Cheng J., Wang Z., Lu Y., Wang J., Wang H. (2022). Weak-solvent-modulated optical encryption based on perovskite nanocrystals/polymer composites. Chem. Eng. J..

[11] Yoon S.J., Draguta S., Manser J.S., Sharia O., Schneider W.F., Kuno M., Kamat P.V. (2016). Tracking Iodide and Bromide Ion Segregation in Mixed Halide Lead Perovskites During Photoirradiation. ACS Energy Lett..

[12] Wu H., Yao L., Cao W., Yang Y., Cui Y., Yang D., Qian G. (2022). Stable and Wide-Wavelength Tunable Luminescence of CsPbX_3_ Nanocrystals Encapsulated in Metal–Organic Frameworks. J. Mater. Chem. C.

[13] Chen T., Xu Y., Xie Z., Jiang W., Wang L., Jiang W. (2020). Ionic Liquid Assisted Preparation and Modulation of the Photoluminescence Kinetics for Highly Efficient CsPbX_3_ Nanocrystals with Improved Stability. Nanoscale.

[14] Zhou Y., Chen J., Bakr O.M., Sun H.-T. (2018). Metal-Doped Lead Halide Perovskites: Synthesis, Properties, and Optoelectronic Applications. Chem. Mater..

[15] Padhiar M.A., Wang M., Ji Y., Yang Z., Zhou Y., Qiu H., Wang H., Shah A.A., Bhatti A.S. (2022). Stable CsPbX_3_ (Br/Cl) Perovskite Nanocrystal Layer Passivated with Al-Doped CdSe for Blue Light-Emitting Diodes. ACS Appl. Nano Mater..

[16] Otero-Martínez C., Fiuza-Maneiro N., Polavarapu L. (2022). Enhancing the Intrinsic and Extrinsic Stability of Halide Perovskite Nanocrystals for Efficient and Durable Optoelectronics. ACS Appl. Mater. Interfaces.

[17] Lou S., Xuan T., Wang J. (2019). (INVITED) Stability: A Desiderated Problem for the Lead Halide Perovskites. Opt. Mater. X.

[18] Hu H., Wu L., Tan Y., Zhong Q., Chen M., Qiu Y., Yang D., Sun B., Zhang Q., Yin Y. (2018). Interfacial Synthesis of Highly Stable CsPbX_(3)_/Oxide Janus Nanoparticles. J. Am. Chem. Soc..

[19] Chen W., Hao J., Hu W., Zang Z., Tang X., Fang L., Niu T., Zhou M. (2017). Enhanced Stability and Tunable Photoluminescence in Perovskite CsPbX_3_/ZnS Quantum Dot Heterostructure. Small.

[20] Huang S., Li Z., Kong L., Zhu N., Shan A., Li L. (2016). Enhancing the Stability of CH3NH3PbBr3 Quantum Dots by Embedding in Silica Spheres Derived from Tetramethyl Orthosilicate in “Waterless” Toluene. J. Am. Chem. Soc..

[21] Jiang M.-C., Lin C.-Q., Yang Z., Pan C.-Y. (2023). Silica-Coated CsPbBr_3_ Nanocrystals with High Stability for Bright White-Emitting Displays. J. Solid-State Chem..

[22] Kumar A., Tripathi S.K., Shkir M., Alqahtani A., AlFaify S. (2023). Prospective and Challenges for Lead-Free Pure Inorganic Perovskite Semiconductor Materials in Photovoltaic Application: A Comprehensive Review. Appl. Surf. Sci. Adv..

[23] Gahlot K., di Mario L., Bosma R., Loi M.A., Protesescu L. (2024). Air-Stable Thin Films of Tin Halide Perovskite Nanocrystals by Polymers and Al_2_O_3_ Encapsulation. Chem. Mater..

[24] Zhong Q., Cao M., Hu H., Yang D., Chen M., Li P., Wu L., Zhang Q. (2018). One-Pot Synthesis of Highly Stable CsPbBr_3_@SiO_2_ Core–Shell Nanoparticles. ACS Nano.

[25] Padhiar M.A., Ji Y., Wang M., Pan S., Khan S.A., Khan N.Z., Zhao L., Qin F., Zhao Z., Zhang S. (2023). Sr^2+^ Doped CsPbBrI_2_ Perovskite Nanocrystals Coated with ZrO_2_ for Applications as White LEDs. Nanotechnology.

[26] Das S., De A., Samanta A. (2020). Ambient Condition Mg^2+^ Doping Producing Highly Luminescent Green- and Violet-Emitting Perovskite Nanocrystals with Reduced Toxicity and Enhanced Stability. J. Phys. Chem. Lett..

[27] Chen C., Xuan T., Bai W., Zhou T., Huang F., Xie A., Wang L., Xie R.-J. (2021). Highly Stable CsPbI_3_:Sr^2+^ Nanocrystals with Near-Unity Quantum Yield Enabling Perovskite Light-Emitting Diodes with An External Quantum Efficiency of 17.1%. Nano Energy.

[28] Yao J.-S., Ge J., Han B.-N., Wang K.-H., Yao H.-B., Yu H.-L., Li J.-H., Zhu B.-S., Song J.-Z., Chen C. (2018). Ce^3+^-Doping to Modulate Photoluminescence Kinetics for Efficient CsPbBr_3_ Nanocrystals Based Light-Emitting Diodes. J. Am. Chem. Soc..

[29] Xu L., Yuan S., Zeng H., Song J. (2019). A Comprehensive Review of Doping in Perovskite Nanocrystals/Quantum Dots: Evolution Of Structure, Electronics, Optics, And Light-Emitting Diodes. Mater. Today Nano.

[30] Ahmad I., Abohashrh M., Aftab A., Aziz H., Fatima I., Shahzadi N., Ahmad S., Muhmood T. (2023). Manganese and Copper Doped Perovskites Nanocrystals and Their Optoelectronic Applications. Appl. Mater. Today.

[31] Zhao Q., Chen F., Huang Q., Wang K., Li C., Wang R., Liu C., Xu W., Liu R., Zhu H. (2024). Shape-Controlled Synthesis of CsPbBr_3_ Nanorods with Bright Pure Blue Emission and High Stability. J. Mater. Chem. C.

[32] Li L., Zhang Z. (2022). In-situ fabrication of Cu doped dual-phase CsPbBr_3_–Cs_4_PbBr_6_ Inorganic Perovskite Nanocomposites for Efficient and Selective Photocatalytic CO_2_ reduction. Chem. Eng. J..

[33] Padhiar M.A., Zhang S., Qin F., Wang M., Ji Y., Khan N.Z., Muhammad N., Khan S.A., Ahmed J., Pan S. (2024). Lead-Free Cs₂NaInCl₆:Bi^3^⁺/Mn^2^⁺ Double Perovskite Nanocrystals to Nanosheets with Improved Photoluminescence Quantum Yield for Anti-Counterfeit Marks And Led Applications. Ceram. Int..

[34] Ornelas-Cruz I., Santos R.M.D., González J.E., Lima M.P., Da Silva J.L.F. (2024). Cubic-to-Hexagonal Structural Phase Transition in Metal Halide Compounds: A DFT study. J. Mater. Chem. A.

[35] Zhang B., Liang Q., Yong X., Wu H., Chu Z., Ma Y., Brovelli S., Manna L., Lu S. (2023). Facet-Defect Tolerant Bi-Doped Cs_2_Ag_x_Na_1–x_InCl_6_ Nanoplatelets with a Near-Unity Photoluminescence Quantum Yield. Nano Lett..

[36] Chan W.K., Zhou D., Yu Z., Tan T.T.Y. (2021). Mechanistic studies of CsPbBr_3_ superstructure formation. J. Mater. Chem. C.

[37] Ghorai A., Singh S., Roy B., Bose S., Mahato S., Mukhin N., Jha P., Ray S.K. (2025). Suppression of Light-Induced Phase Segregations in Mixed Halide Perovskites Through Ligand Passivation. J. Phys. Chem. Lett..

[38] Yang J., Liu Y., Cai Y., Zhang Y., Zhou P., Liu B., Li Y. (2024). Phase Stability and Electronic Structure of CsPbBr_3_ Perovskites Under Rare-Earth Doping and Hydrostatic Pressure. J. Mater. Sci..

[39] Zeng Y.-T., Li Z.-R., Chang S.-P., Ansay A., Wang Z.-H., Huang C.-Y. (2022). Bright CsPbBr_3_ Perovskite Nanocrystals with Improved Stability by In-Situ Zn-Doping. Nanomaterials.

[40] Keeble D.J., Wiktor J., Pathak S.K., Phillips L.J., Dickmann M., Durose K., Snaith H.J., Egger W. (2021). Identification of Lead Vacancy Defects in Lead Halide Perovskites. Nat. Commun..

[41] Varnakavi N., Gupta K., Lee K., Yang J., Cha P.-R., Lee N. (2023). Compositional Engineering of ZnBr_2_-Doped CsPbBr_3_ Perovskite Nanocrystals: Insights into Structure Transformation, Optical Performance, and Charge-Carrier Dynamics. J. Mater. Chem. C.

[42] Mączka M., Sieradzki A., Bondzior B., Dereń P., Hanuza J., Hermanowicz K. (2015). Effect of Aliovalent Doping on the Properties of Perovskite-Like Multiferroic Formates. J. Mater. Chem. C.

[43] Luo L., Hu W., Liang X., Ding Y., Yuan H., Song Y., Deng S., Kang K. (2024). Study of Phase Transition, Structural Stability and Mechanical Properties of CsPbBr_3_ Under High Pressure by First Principles. Mater. Today Commun..

[44] Xie S., Yang D., Li Z., Ma X., Wang H., Liu S., Liu Y., Yue S. (2025). The Evolution of Electrical, Optical, and Mechanical Properties of CsPbBr_3_ Perovskites During Continuous Phase Transitions. Chem. Eng. J..

[45] Lawal S.O., Takahashi Y., Nagasawa H., Tsuru T., Kanezashi M. (2022). Microporous Structure Control of SiO_2_-ZrO_2_ Composite Membranes via Yttrium Doping and an Evaluation of Thermal Stability. J. Sol-Gel Sci. Technol..

[46] Chen R., Xu Y., Wang S., Xia C., Liu Y., Yu B., Xuan T., Li H. (2021). Zinc Ions Doped Cesium Lead Bromide Perovskite Nanocrystals with Enhanced Efficiency and Stability for White Light-Emitting Diodes. J. Alloys Compd..

[47] Guo X., Lv Y., Wang L., Liu H., Wang W. (2024). CsPbBr_3_ Perovskite Quantum Dots by Mesoporous Silica Encapsulated for Enhancing Water and Thermal Stability via High Temperature Solid State Method. Opt. Mater..

[48] Cai Y., Wang L., Zhou T., Zheng P., Li Y., Xie R.-J. (2018). Improved Stability of CsPbBr_3_ Perovskite Quantum Dots Achieved by Suppressing Inter ligand Proton Transfer and Applying a Polystyrene Coating. Nanoscale.

[49] Schryver S., Lamichhane A. (2023). Temperature-driven structural phase transitions in CsPbBr_3_. Solid State Commun..

[50] Li Y., Dong L., Patterson R., Teh Z.L., Hu Y., Huang S., Chen C. (2020). Stabilizing CsPbBr_3_ perovskite quantum dots on zirconium phosphate nanosheets through an ion exchange/surface adsorption strategy. Chem. Eng. J..

[51] Yan D., Zhao S., Zhang Y., Wang H., Zang Z. (2022). Highly efficient emission and high-CRI warm white light-emitting diodes from ligand-modified CsPbBr_3_ quantum dots. Opto-Electron. Adv..

[52] Rao L., Sun B., Liu Y., Zhong G., Wen M., Zhang J., Fu T., Wang S., Wang F., Niu X. (2023). Highly Stable and Photoluminescent CsPbBr_3_/Cs_4_PbBr_6_ Composites for White-Light-Emitting Diodes and Visible Light Communication. Nanomaterials.

[53] Li Y., Lv Y., Guo Z., Dong L., Zheng J., Chai C., Chen N., Lu Y., Chen C. (2018). One-Step Preparation of Long-Term Stable and Flexible CsPbBr_3_ Perovskite Quantum Dots/Ethylene Vinyl Acetate Copolymer Composite Films for White Light-Emitting Diodes. ACS Appl. Mater. Interfaces.

[54] Guan H., Zhao S., Wang H., Yan D., Wang M., Zang Z. (2020). Room temperature synthesis of stable single silica-coated CsPbBr_3_ quantum dots combining tunable red emission of Ag–In–Zn–S for High-CRI white light-emitting diodes. Nano Energy.

