# Supplementary material for: ZrBr4-Mediated Phase Engineering in CsPbBr3 for Enhanced Operational Stability of White-Light-Emitting Diodes
Source: Nanomaterials (Basel). 2025 Apr 28;15(9):674. doi: 10.3390/nano15090674 (PMC12073312; doi:10.3390/nano15090674)
Supplement: Supplementary file 1 [file nanomaterials-15-00674-s001.zip › nanomaterials-3591105-supplementary.pdf]

$ZrBr_4$-Mediated Phase Engineering of $CsPbBr_3$ for Enhanced operational Stability of White LEDs

Muhammad Amin Padhiar [a,b], Yongqiang Ji [c*], Jing Wang [a], Noor Zamin Khan [b], Mengji Xiong [a] Shuxin Wang [a*]

E-mail: amin.padhiar@gzhu.edu.cn, yongqiangji@pku.edu.cn, wangsx@shzq.edu.cn

[a] School of Intelligent Manufacturing, Shanghai Zhongqiao Vocational and Technical University, Shanghai, China

[b] School of Physics and Materials Science, Guangzhou University, Guangzhou 510006, China.

[c] Institute of Physics, Henan Academy of Sciences Zhengzhou, 450046, China.

1. Experimental Section

1.1. Materials

Cesium carbonate (Cs_2CO_3, 99.99%), Zirconium (IV) bromide ($ZrBr_4$, 99.99%), Lead (II) bromide ($PbBr_2$, 99.99%), Oleic acid (OA, 85%), Oleylamine (OA, 80~90%), 1-Octadecene (ODE, 90%), and Toluene (>99.7%) were purchased from Aladdin. LED 450 nm blue light chip were purchased from CREE company. All the reagents were used without further purification.

1.2 Synthesis of Cs- Oleate

2.5 mmol Cs_2CO_3 was placed in solution consisting of 2.5 mL OA and 10 mL ODE and transferred to 100 mL three-neck flask. The solution was heated to 120 °C under vacuum and reacted for 1 h. After that, the Cs-OA precursor was obtained by heating to 120 °C and reacting for 1 h under N_2 environment. Note that the Cs-OA precursor needs to be held at 100 °C before use.

1.3 Synthesis of $CsPBr_3$ NCs

The $CsPbBr_3$ NCs were synthesized via the hot injection method, as previously reported in our work [1], with slight modifications. The synthesis of process of $CsPbBr_3$ NCs was initiated by adding 0.46 mmol (0.13 g) of $PbBr_2$ to a 100 ml three-neck flask containing 10 ml of ODE. The mixture was then stirred at 120 °C to ensure thorough mixing. Following this, 1 ml of OLA, 1 ml of OA were injected into the flask to facilitate the reaction. The temperature of the reaction mixture was subsequently increased to 170 °C to initiate the formation of NCs. Finally, 0.8 ml of a preheated Cs-oleate solution was added to the mixture. After the reaction, the solution containing the NCs was gradually cooled using a water bath.

1.4 Synthesis of $ZrBr_4$ Oleate

Dissolve $ZrBr_4$ in varying amounts— 0.12 g (0.25 mmol), 0.23 g (0.5 mmol), 0.46 g (1 mmol), and 0.92 g (2 mmol), in a mixture of 2 mL ODE and 0.5 mL OA under a N_2 atmosphere at 80°C until the salts were completely dissolved. The synthesis was controlled according $PbBr_2$:$ZrBr_4$ ratios into 1:0.25, 1:50, 1:1 and 1:2 respectively as mention in the below Table S1.

Table S1. composition of each element during synthesis process

**Molar Concentration**	**$PbBr_2$ (mmol)**	**$ZrBr_4$ (mmol)**	**Cs-OA (ml)**	***x* ($PbBr_2$:$ZrBr_4$ Ratio)**
1:0.25	0.46	0.25	0.9	x= 0.25
1:50	0.46	0.50	0.9	x= 0.5
1:1	0.46	1	0.9	x= 0.1
1:2	0.46	2	0.9	x= 0.2

1.5 Synthesis of $ZrBr_4$:$CsPbBr_3$ HNs

After synthesizing the $CsPbBr_3$ NCs, allows the reaction to cool to 90 °C. At this temperature, inject the pre-prepared $ZrBr_4$ oleate solutions into the mixture. Maintain this temperature and stir the solution for a predetermined duration to facilitate the desired phase transformation. Monitor the transformation by collecting samples at various time intervals, as detailed in (Table S2). Once the phase transformation is complete, cool the mixture to room temperature. Isolate the precipitates by centrifuging the solution at 1000 rpm for 5 minutes. Subsequently, wash the precipitates 2 times with toluene to purify the final product.

Table S2. The phase transformation stages

**Reaction Time**	**Phase transformation**	**Rationale**
10 mis	Partial hexagonal phase (~40%) + residual cubic phase	Limited time for Zr^{4+} diffusion and lattice reorganization.
20 mins	Dominant hexagonal phase (~70%)	Extended time enhances $ZrBr_4$-mediated stabilization and reduces cubic defects.
30 mins	Fully hexagonal phase (~98–100%)	Complete lattice rearrangement and Zr^{4+} incorporation.

1.6 Synthesis of $CsPBrbI_2$

0.5 mmol of PbI_2 and $PbBr_2$was placed in solution consisting of 10 mL ODE, 1 mL OA

and 1 mL OAm and transferred to 100 mL three-neck flask. The temperature was increased to 120 °C for 1 h under N_2, and then increased to 150 °C to continue reaction until salts were completely dissolved. Finally, 0.8 ml of a preheated Cs-oleate solution was added to the mixture. After the reaction, the solution containing the NCs was gradually cooled using a water bath.

1.7 Preparation of white light-emitting diodes ($ZrBr_4$-WLED).

The synthesized green HNs, exhibiting the highest PLQY $CsPbBr_3$:$_x$$ZrBr_4$, ($x$= 0.1) along with red-emitting $CsPbBrI_2$, were applied onto flexible polyethylene terephthalate (PET) films using the doctor blade technique. The coated PET films were then cut to appropriate dimensions and affixed to a blue light source to evaluate their performance as light-emitting diodes (LEDs)

1.8 Characterization

The morphology of the as-prepared NCs and HNs was examined using high-resolution transmission electron microscopy (JEOL JEM-F200). Their chemical composition was determined with an energy-dispersive spectrometer (EDS, Oxford X-Max 65) integrated into the TEM. The X-ray diffraction (XRD) patterns were recorded with a DB-ADVANCE instrument, and X-ray photoelectron spectroscopy (XPS) analysis was conducted using a Thermo Fisher ESCALA670B Xi^+. Ultraviolet–visible (UV–vis) absorption spectra were measured with a PE Lambda 950, while photoluminescence (PL) spectra, PLQYs, and time-resolved PL (TRPL) decay curves were collected using an Edinburgh Instruments FLS980 spectrometer. The PLQY was measured by using the FLS980 spectrometer, which performs absolute PLQY measurements using an integrating sphere. This method provides a direct measurement of the total number of photons emitted by the sample relative to the total number of photons absorbed. Device performance was evaluated using a PR-670 spectrometer.

1.9 TRPL Decay Curves Fitting

The PL decay curves were calculated with a bi-exponential decay model as presented equations (S1-S2). The bi-exponential decay equation describes the time-resolved TRPL decay of the samples. It consists of two exponential components to fit the data, as the PL decay in these samples may follow more than one process with different time constants.

Whereas, *I*: The intensity of the PL as a function of time (t). B_1: The pre-exponential factor for the fast decay component. This corresponds to the contribution of the excitonic radiative recombination process in the sample. B_2: The pre-exponential factor for the slow decay component. This corresponds to the contribution of surface radiative defect recombination in the sample. τ_1: The decay time constant for the fast decay component, associated with the excitonic radiative recombination. τ_2: The decay time constant for the slow decay component, associated with surface radiative defect recombination.

$$I = B_1\, exp\left(-\frac{t}{\tau_1}\right) + B_2\, exp\left(-\frac{t}{\tau_2}\right) \qquad (S1)$$

$$\tau_{avu} = \frac{B_1\tau_1^2 + B_2\tau_2^2}{B_1\tau_1 + B_2\tau_2} \qquad (S2)$$

Table S3 Bi-exponential fitting results of the time resolved PL spectra of $CsPbBr_3$:$ZrBr_4$ (*x*=0, 0.25, 0.5,0.1 and 0.2) respectively

***x* = 0**	***x* = 0.25**	***x* = 0.5**	***x* = 0.1**	***0.2***
τ_1=19 ns	τ_1=29 ns	τ_1=37 ns	τ_1=50 ns	τ_1=44 ns
B_1%=73.9	B_1%=78.5	B_1%=80.6	B_1%=100	B_1%=94
τ_2=20.6 ns	τ_2=56.8 ns	τ_2=68 ns	τ_2=74 ns	τ_2=69 ns
B_2%=27.9<br>τ_{avg}=19.7 ns	B_2%=28.4<br>τ_{avg}=38.83 ns	B_2 %=36<br>τ_{avg}= 54.28 ns	B_2%=39<br>τ_{avg}=78.86 ns	B_2%=35<br>τ_{avg}=65.51 ns

References

1. M.A. Padhiar, Y. Ji, M. Wang, S. Pan, S. Ali khan, N.Z. Khan, L. Zhao, F. Qin, Z. Zhao, S. Zhang, Sr^{2+} Doped $CsPbBrI_2$ Perovskite Nanocrystals Coated with ZrO_2 for Applications as White LEDs, Nanotechnology, 34 (2023) 275201.
